# Distribution and Biology of *Protaetia fieberi* (Coleoptera, Scarabaeidae)—Is Protection Status Required?

**DOI:** 10.3390/insects15090695

**Published:** 2024-09-13

**Authors:** Leonid V. Egorov, Alexander B. Ruchin, Anatoliy A. Khapugin

**Affiliations:** 1 Prisursky State Nature Reserve, 428034 Cheboksary, Russia; platyscelis@mail.ru; 2 Joint Directorate of the Mordovia State Nature Reserve and National Park “Smolny”, 430005 Saransk, Russia; hapugin88@yandex.ru; 3 Institute of Environmental and Agricultural Biology (X-BIO), Tyumen State University, 625003 Tyumen, Russia

**Keywords:** *Protaetia fieberi*, region, biotope, saproxylic beetles, distribution area, European Russia

## Abstract

**Simple Summary:**

The distribution and biology of *Protaetia fieberi* (Kraatz, 1880) (Scarabaeidae: Cetoniinae) in European Russia were investigated. This species is reliably known from 26 regions. The limiting factors that prevent the spread to the north, south, and southeast of European Russia have been identified. Within its range, the species inhabits deciduous and mixed forests of various types. The species also inhabits anthropogenic forest ecosystems. In forests, the species adheres to the upper tiers of the forest. Adults feed on the leaking sap on trunks and less often on flowering plants. Seasonal dynamics is characterized by highs from the third decade of June to the second decade of July. The status of the species is being discussed.

**Abstract:**

Studies on saproxylic species of Coleoptera have garnered significant attention due to the rarity of some of them. To investigate the distribution and biology of *Protaetia fieberi* (Kraatz, 1880) (Scarabaeidae: Cetoniinae) in European Russia, we analyzed data from 16 regions collected between 2018 and 2024. This species has been reliably recorded in 26 regions. We describe the species’ distribution area boundaries and discuss limiting factors that inhibit its spread to the north, south, and southeast of European Russia. The primary limiting factor is the lack of suitable biotopes. Within its distribution, *Protaetia fieberi* prefers deciduous and mixed forests of various types. These habitats include both old-growth forest ecosystems and secondary forests that have regenerated following logging. The species also inhabits man-made forest ecosystems, such as field protection forest belts, old parks, and gardens. In forest ecosystems, *Protaetia fieberi* tends to occupy the upper levels and is rarely found on the ground layer. Conversely, in open areas such as glades, the species is more commonly found at ground level. This distribution pattern is linked to the adults’ feeding preferences, which include consuming sap on tree trunks in forests and feeding on flowering plants in open ecosystems. The seasonal activity of *Protaetia fieberi* peaks from the third decade of June to the second decade of July. It is hypothesized that the perceived rarity of *Protaetia fieberi* in research samples is due to the specific baiting methods used, with beer traps being the most effective. The status of the species is re-evaluated in light of new data, suggesting that *Protaetia fieberi* is common rather than rare in European Russia.

## 1. Introduction

Forests are subject to various dynamic changes, primarily driven by human activities such as forestry, fires, the introduction of invasive species, and other factors [1,2,3,4,5,6,7]. These factors negatively impact forest biodiversity, including saproxylic organisms—species that depend on the fungal decomposition of wood. Saproxylic organisms include species that feed on wood [8] and those that participate in the decomposition process.

The presence of saproxylic species is significantly influenced by the availability of dead wood, stumps, and large, hollow-leaved trees. These species also require extensive and regularly distributed areas of mature, undisturbed forest [9,10,11,12]. Deciduous and mixed forests are the primary habitats for saproxylic organisms, with coniferous forests playing a lesser role [13,14,15,16,17]. Due to their significant biodiversity, wide distribution of ecological preferences, and role in wood decomposition, saproxylic species are among the most important groups of organisms in forest ecosystems [18,19,20].

The methods for studying saproxylic species of Coleoptera are diverse, employing a distribution area of collection techniques. Traditional methods for collecting saproxylic beetles include direct active collection techniques, rearing techniques, and mass trapping methods [21]. Among the mass trapping methods are light intercepting traps, traps that are the most widely used. These traps are typically installed singly or in groups at a specific height above the ground [11,22]. Emergence traps, which consist of bags wrapped around parts of stumps, deadwood, and tree trunks and connected to jars for collecting emerging insects, are also commonly used [22,23]. Emergence traps are particularly effective, collecting significantly more Coleoptera than single sieving methods [22]. Although sticky traps are used infrequently, they can be effective for studying saproxylic species on individual trees [24]. Sieving of bark, deadwood, and tree stumps is another method used to identify beetle preferences for certain tree species [22,25]. However, this process is time-consuming and not always feasible for large-scale scientific projects. For a comprehensive study of saproxylic species, it is often advisable to use a combination of methods [25,26,27,28,29]. Recently, baits made from beer mixed with sugar, honey, molasses, and other sugar-containing ingredients have been introduced for studying saproxylic species. These baits can help identify not only species diversity but also species abundance, biotope diversity, and seasonal dynamics [15,30,31,32].

The aim of this study is to investigate the distribution and biology of *Protaetia fieberi* (Kraatz, 1880) in European Russia to better understand its conservation status. Additionally, the study aims to compare habitat preferences across various habitat types within the studied distribution area.

## 2. Materials and Methods

Our research was conducted from 2018 to 2024 across 16 regions of the European part of Russia, specifically in the Lipetsk Region, Voronezh Region, Ryazan Region, Tambov Region, Moscow Region, Vladimir Region, Nizhny Novgorod Region, Republic of Mari El, Chuvash Republic, Republic of Mordovia, Republic of Tatarstan, Ulyanovsk Region, Samara Region, Saratov Region, Penza Region, and Volgograd Region. During this period, we investigated over 850 localities, utilizing more than 1100 different traps.

European Russia is primarily situated on the Eastern European Plain, characterized by mostly flat terrain. The mainland includes the subarctic and temperate climatic zones. The extensive plain stretches from north to south creating well-defined zones in the distribution of its landscapes. The region experiences a gradient of climatic conditions from north to south, with increasing dryness. In the north, the forest zone features a climate with excessive moisture, high rainfall, and relatively high humidity. As one moves southward, the forest–steppe zone serves as a transitional area, leading to the steppe and semi-desert climates and is characterized by low precipitation and dry air. Historically, the middle zone was dominated by typical forest landscapes, including dark coniferous taiga, mixed forests, and broadleaved oak and lime forests. However, many forest ecosystems in this area have been adversely affected by human activities.

East of the Lower Volga, the High Volga region is noteworthy, directly adjacent to the Middle and Southern Urals and merging with their foothills. This area is also characterized by extensive lowlands, such as the Caspian Lowland in the southeast, which transitions from steppes to semi-deserts. The southern part of European Russia features steppe landscapes that gradually transition into the foothills and mountains of the Caucasus [33].

Sampling was conducted using a variety of methods, including pitfall traps, light traps, sweep nets, Malaise traps, freely hanging flight-intercept (window) traps, pan traps, and beer traps. Among these, beer traps were especially actively used. These traps, made from plastic bottles with a cut-out window, were baited with a mixture of beer, jam, honey, and sugar to attract beetles [34]. The vertical distribution of *Protaetia fieberi* in forest ecosystems, forest edges, and open biotopes was studied using these beer traps installed at various heights from the soil surface (ranging from 1.5 to 12 m). Sampling continued throughout the beetle activity season, from April to September.

In each location where beer traps were installed, several parameters were recorded (abundance of species, number of days of trap exposure, species composition of trees in all tiers of the forest, crowns, undergrowth, grassy cover, number of dry trees and stumps, predominant species of deadwood and stumps, number and species composition of fallen trees). These included the type of forest (small-leaved, broadleaved, or mixed forest), the number of trapped *Protaetia fieberi* individuals, and the duration of the beer trap exposure (the height of the traps varied from 1.5 to 12 m). When describing the biotope, the percentage covered by the forest stand (1–2 tiers), the undergrowth, and the herb layer were documented. Additionally, the percentage cover and development of deadwood (standing dry ancient plants), stumps, and fallen leaves of ancient plants within the field of visibility were determined. These data were subsequently used for principal component analysis (PCA) of *Protaetia fieberi* habitats. We tested whether there are differences among identified plant communities (forest belt, mixed, small-leaved, and broadleaved forest), based on species abundance and habitat characteristics (mentioned above), which were also used as response variables in PCA. To estimate the differences among the identified plant communities, a one-way Permutational MANOVA (PerMANOVA) test with Bonferroni correction was performed based on the number of collected specimens and habitat characteristics (*p* < 0.001; F = 20.9; total sum of squares = 1.7 × 10^−5^; 9999 permutations). Using the similarity percentage method (SIMPER), we identified response variable factors responsible for the dissimilarity (Bray–Curtis matrix) of the plant communities identified. All statistical analyses were conducted using PAST 4.11 software [35].

When mapping the distribution and abundance of *Protaetia fieberi*, a daily grid map with a cell area of approximately 2500 km^2^ was used. The abundance of the species within each cell was categorized as follows: *single individual* indicates that only single specimens were found in 1–2 localities within the cell, *rare species* indicates that no more than 15 specimens were found in 5–10 localities within the cell, and *common species* indicates that at least 100 specimens were found in more than 11 localities within the cell.

## 3. Results

Within European Russia, the northern border of the *Protaetia fieberi* distribution area extends through the Ivanovo, Nizhny Novgorod, and Kirov regions, as well as the Udmurt Republic. The eastern boundary of the known distribution encompasses the Republic of Tatarstan, Udmurtia, Bashkiria, and the Orenburg region. South of the Don River in the Rostov region, *P. fieberi* has not been observed. In the Caucasus, the species has only been recorded in Novorossiysk [36,37]. To the south, *Protaetia fieberi* persists in small forested areas predominantly composed of deciduous species.

**Figure 1:**
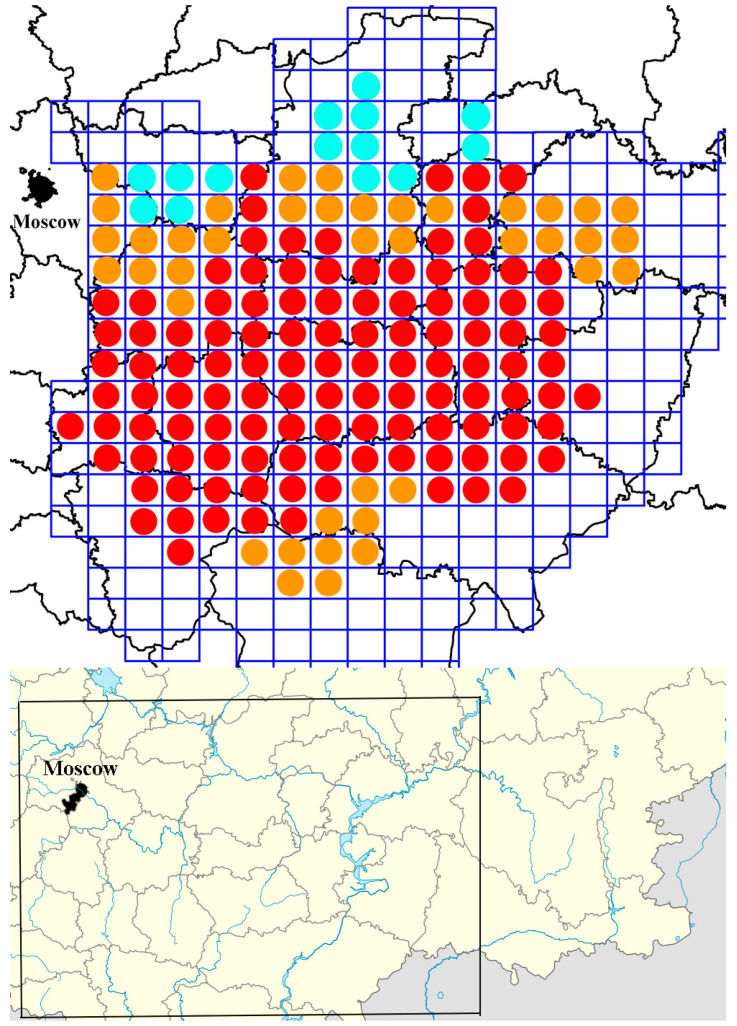
An approximate estimate of the number of *Protaetia fieberi* in European Russia according to the data collected within the present research from 2018 to 2023. Red circles show cells (50 × 50 km) where *Protaetia fieberi* is common in localities; orange circles indicate cells where *Protaetia fieberi* is rare in localities; blue circles indicate cells where *Protaetia fieberi* is rare in localities.

The eastern boundary of the distribution area is likely influenced not only by the limited number of suitable biotopes but also by abiotic factors. The species is most commonly found south of 54°54′ N latitude. North of this boundary, *Protaetia fieberi* becomes rarer, and north of the Volga River, sightings are isolated (Figure 1). An increase in the number and occurrence of the species is observed in forest ecosystems located in the floodplains of large rivers, such as the Volga, Sura, and Oka. The primary limiting factors for *Protaetia fieberi* populations include the rarity and vulnerability of the species at the northern and eastern borders of its distribution area, the reduction in areas occupied by mature and overgrown oak forests, and the decline and felling of old oaks.

**Figure 2:**
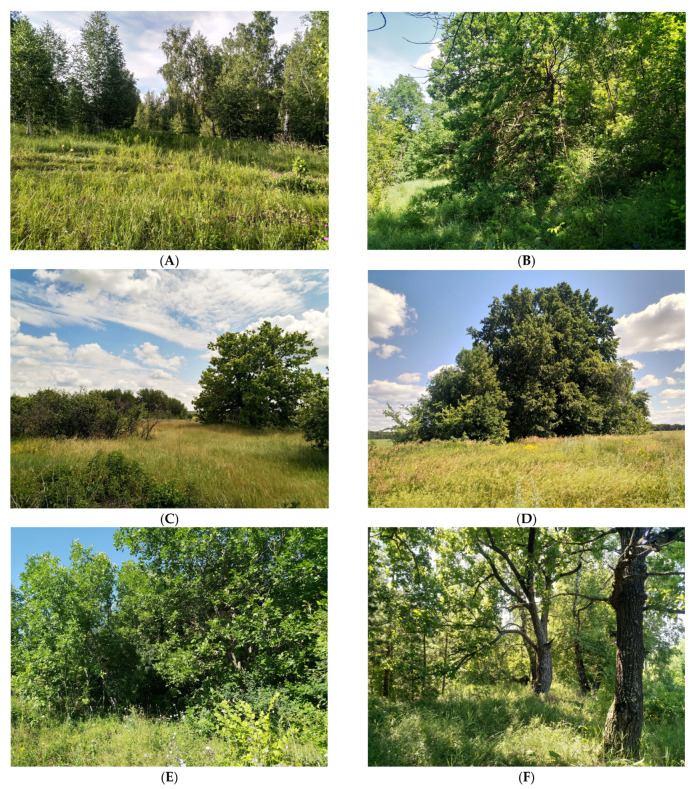
Preferred biotopes of *Protaetia fieberi*. (**A**)—Saratov Region, Atkarsk district, Peschanka; (**B**)—Saratov Region, Novye Burassy district, Teplovka; (**C**)—Tambov Region, Uvarovo district, Perevoz; (**D**)—Voronezh Region, Gribanovo district, Bolshye Alabukhi; (**E**)—Samara Region, Syzran district, Zaborovka; (**F**)—Ulyanovsk Region, Kuzovatovo district, Privolie.

Figure 2 illustrates these findings, showing the various habitats and the distribution of *Protaetia fieberi* within these ecosystems. These data highlight the species’ adaptability to different forest types and its ability to thrive in both natural and human-modified environments.

Populations of *Protaetia fieberi* are found in a diverse distribution area of forest ecosystems, both natural and anthropogenic. Most of the specimens we studied were collected in various types of broadleaved forests (including floodplain oaks, deciduous forests of overgrown origin, forests with linden and maple, and aspen forests) and mixed forests (primarily complex pine forests with a significant percentage cover of deciduous trees in the first and second tiers). Occasionally, the species was found in complex pine forests with a well-developed second tier of broadleaf, such as in the Republic of Mordovia and the Republic of Tatarstan.

In the Voronezh, Tambov, Lipetsk, Samara, Ulyanovsk, and Saratov regions, *Protaetia fieberi* was consistently recorded in protective forest belts composed of oak, poplar, linden, birch, and maple, with occasional ash (Figure 2). In Chuvashia, the species was repeatedly noted in old apple orchards. The species was also frequently recorded in traps placed on isolated trees far from large forest ecosystems, indicating its strong dispersal ability. In the Orenburg region, *Protaetia fieberi* was found in broadleaved forests, showing a preference for areas with oak and linden [38].

**Figure 3:**
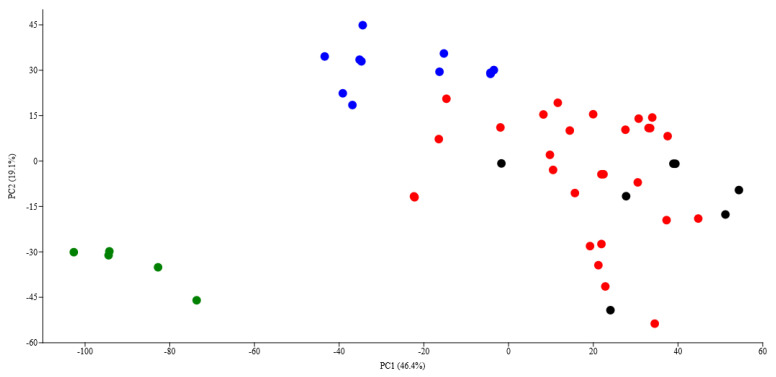
Principal component analysis of locations of *Protaetia fieberi* based on the number of specimens and habitat characteristics in nine regions of European Russia. Designations: blue colour—mixed forests, black colour—forest belt plantations, red colour—broadleaved forests, green colour—small-leaved forests.

One-way perMANOVA for differences in species abundance and habitat characteristics between plant communities (forest belts, mixed, broadleaved, and small-leaved forests) revealed that small-leaved forests differ significantly from forest belts (*p* < 0.05), mixed (*p* < 0.001) and broadleaved (*p* < 0.001) forests. There were also significant differences between mixed forests and both forest belts (*p* < 0.001) and broadleaved forests (*p* < 0.001). There was no significant difference (*p* = 0.2) between broadleaved forests and forest belts. These results are confirmed by PCA, where the locations of the forest belts and small-leaved forests are situated separately from other locations on the plot (Figure 3).

Similarity percentage analysis (SIMPER) revealed that the percentage cover of oak, pine, and birch stand species, as well as grasses and herbs of the herb layer, contributed most significantly (50.8% in total) to the differences among three types of biotopes:

- Oak of the 1st tier (average dissimilarity = 6.2, % contribution = 16.2%);
- Birch of the 1st tier (average dissimilarity = 4.1, % contribution = 10.6%);
- Pine of the 1st tier (average dissimilarity = 3.5, % contribution = 9.0%);
- Birch undergrowth (average dissimilarity = 3.1, % contribution = 8.0%);
- Cereals (average dissimilarity = 3.0, % contribution = 7.7%);
- Different grasses (average dissimilarity = 2.8, % contribution = 7.5%).

The participation of these elements in the plant communities varied markedly across different localities, yet all these components are crucial for communities inhabited by *Protaetia fieberi*. Interestingly, species abundance (number of collected species) did not significantly contribute to the differentiation of localities.

Correlation analysis further indicated that there was no significant dependence (r < 0.38, *p* > 0.05) of the number of captured *Protaetia fieberi* specimens on most biotope characteristics. However, a weak significant relationship was found between the percentage cover of oak undergrowth and the number of *Protaetia fieberi* specimens (r = 0.378, *p* = 0.006).

**Figure 4:**
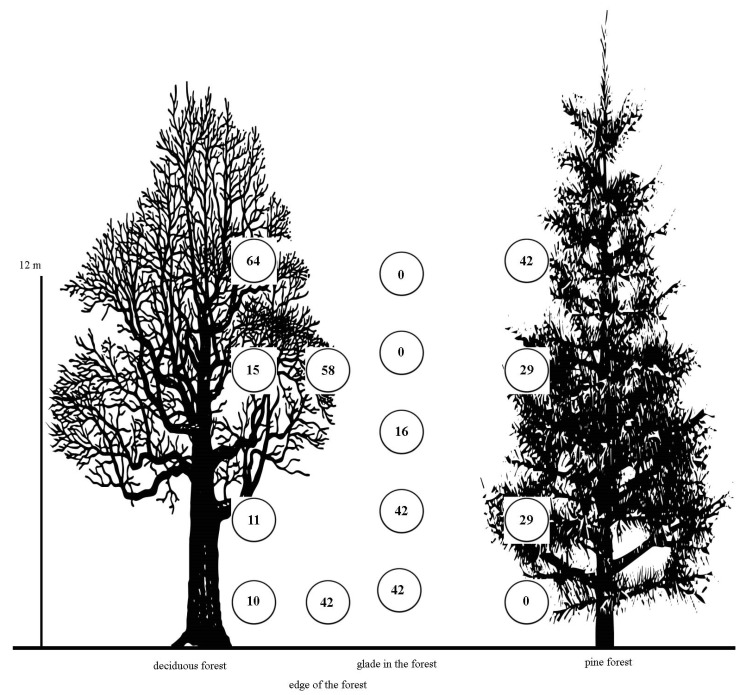
Numerical distribution of *Protaetia fieberi* in forest ecosystems and in forest clearings of European Russia at different heights from the surface. The height from the ground level is indicated on the left. The circles indicate the number of *Protaetia fieberi* at a certain height (as a percentage of the number of captured individuals).

Figure 4 presents the results of a study on the vertical distribution of *Protaetia fieberi* in both forest and non-forest ecosystems in European Russia. In forest ecosystems, the species shows a preference for the upper tiers, with the highest numbers observed in the tree crowns. Below the tree crowns, in bushes and shrubs, the number of individuals decreases significantly [12]. In contrast, in non-forest ecosystems, *Protaetia fieberi* distribution tends to be more even, with a noticeable presence at the level of the undergrowth and herb layer [39].

**Figure 5:**
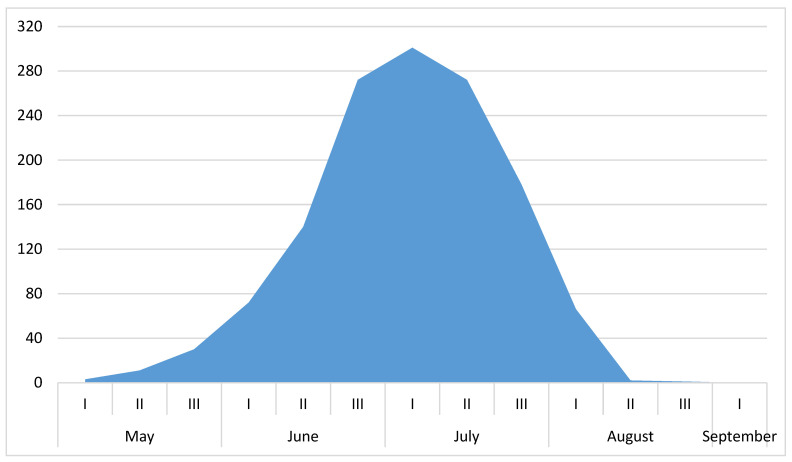
Seasonal dynamics of *Protaetia fieberi* in the European part of Russia (based on observations of 1348 specimens).

Our analysis of both our own and published data on the phenology of adult *Protaetia fieberi* in European Russia reveals that the main period of imago activity occurs from the third decade of June to the second decade of July (Figure 5). The first individuals are typically observed in the first days of May, with the last imago of *Protaetia fieberi* noted at the end of August. In September, adults are not encountered. This phenological pattern is consistent with observations in other regions, such as France [40] and Hungary [41], where similar periods of activity have been reported.

The feeding behaviour of *Protaetia fieberi* imago has been well studied across different parts of its distribution area. It has been observed that individuals are particularly attracted to the leaking sap of various trees, predominantly oaks. In addition to tree sap, adult *Protaetia fieberi* also feeds on a variety of flowering plants belonging to the families Umbelliferae, Asteraceae, and Rosaceae [38,40,42,43,44].

## 4. Discussion

In European Russia, *Protaetia fieberi* is widespread in the forest–steppe, partly in steppe zones, and in the Pre-Caucasus region. Reliable findings have been documented in the following 26 regions [44,45,46,47,48]: Belgorod, Vladimir, Volgograd, Voronezh, Ivanovo, Kaluga, Kirov, Lipetsk, Moscow, Nizhny Novgorod, Orenburg, Penza, Rostov, Ryazan, Samara, Saratov, Tambov, Tula, Ulyanovsk regions, Republic of Mari El, Republic of Mordovia, Udmurt Republic, Republic of Bashkortostan, Republic of Tatarstan, Chuvash Republic, and Krasnodar Territory.

Previously, it was believed [49] that the northern border of *Protaetia fieberi’s* distribution in the European part of Russia passed through the south of the Tula region, extending through Penza and Samara, with the eastern border following the Volga Valley to Kamyshin (Volgograd region). The southern border was thought to run along the southern edge of the Voronezh Region and towards the middle course of the Seversky Donets River. However, recent data indicate that the distribution area is much wider, especially towards the north and east [45].

The distribution of *Protaetia fieberi* in European Russia appears to be confined to regions with forest ecosystems or forested areas suitable for its habitat. Birch forests of secondary origin, which grow across the Volga River in the Ivanovo and Nizhny Novgorod regions, as well as in the north of the Ryazan region and most of the Vladimir region, form part of its distribution area. Additionally, some isolated patches of oak and linden forests can be found in floodplains, but these areas are scattered and lack connectivity. This fragmentation makes it unlikely for *Protaetia fieberi* to complete its entire life cycle in these regions, thereby limiting its distribution to suitable habitats.

Further north, the landscape changes with the appearance of spruce forests, leading to a decline in large broadleaved forest areas. In Udmurtia, most sightings of *Protaetia fieberi* are in old deciduous forests within the valleys of the Kama and Vyatka rivers, with the largest population in floodplain broadleaved forests [50]. Thus, north of the species’ main distribution area (north of 56°16′ N latitude), *Protaetia fieberi* is primarily found in floodplain deciduous forests. It is likely that the presence of these forest ecosystems has facilitated the species’ northward spread. This pattern of distribution in floodplain broadleaf forests is also observed in Hungary [42].

In the southern part of *Protaetia fieberi*’s distribution area, the forest–steppe transitions into the steppe zone, and the species inhabits isolated forest areas. These deciduous forests are typically of secondary origin and are often surrounded by agroecosystems. However, these isolated forests are frequently connected by protective forest belts, which create corridors that facilitate the species’ habitat and migration. In the absence of large forest ecosystems, *Protaetia fieberi* appears to persist in these anthropogenic habitats.

Further south, in the Volgograd and Rostov regions, there are very few remaining forest areas. The protective forest belts in these regions are characterized by relatively young trees, which limit the availability of old, hollow trees necessary for the species’ reproduction. Additionally, these forest belts are often situated in areas with strong winds and poor soil conditions, resulting in stunted trees with thin trunks that are unsuitable for the development of *Protaetia fieberi* larvae.

Typically, when characterizing the biotopes preferred by *Protaetia fieberi*, researchers indicate oak forests. For instance, in Spain, this species is commonly found in old oak groves [51]. In Hungary, *Protaetia fieberi* has been found not only on oaks but also on willows and poplars, with significant numbers observed in old oak forests [41,42]. Conversely, in Romania, the abundance of this species in mixed forests was extremely low [52]. In Germany, the species is found only in a few localities, characterized by deciduous forests with a predominance of oaks [53].

Our analysis of preferred biotopes did not reveal significant preferences for *Protaetia fieberi*. Its abundance did not vary significantly among the studied biotopes, which included mixed forests, forest belt plantations, broadleaved forests, and small-leaved forests. Correlation analysis indicated the absence of a significant dependence on the number of captured specimens on almost all biotope characteristics, including the species composition of trees in the first and second tiers, as well as the presence of dead wood. Notably, *Protaetia fieberi* was found even in forests where birch was the predominant species, and oaks were absent.

We believe that the continuous distribution of *Protaetia fieberi* across the studied regions of European Russia (Figure 1), combined with its significant presence in diverse ecosystems, suggests that the species does not exhibit strong preferences for specific breeding sites. Instead, its habitat suitability appears to be determined by the availability of various deciduous tree species within these ecosystems.

Numerous publications have highlighted oaks, willows, poplars, and linden trees as key sites for larval development of *Protaetia fieberi* [36,37,41,42,49]. Tauzin [40] compiled a broad distribution area of studies, indicating that *P. fieberi* larvae develop in a variety of old-growth trees, including oaks (*Quercus ilex*, *Quercus suber*, *Quercus robur*, *Quercus humilis*, and *Quercus petraea*), beech, several species of willows (*Salix* spp.), chestnut, apple, linden, poplar, white acacia, and mulberry. This extensive distribution area of host trees underscores the species’ adaptability to different types of deciduous trees for larval development.

The occurrence of *Protaetia fieberi* in the upper tiers of forest ecosystems is quite consistent. We believe that in deciduous forests, individuals of this species preferentially occupy the tree crowns due to their feeding behaviour, particularly on the juice exuded from tree trunks. To a lesser extent, *Protaetia fieberi* is an anthophile [38,54,55]. The shrub layer in deciduous forests is typically underdeveloped, and the herb layer is sparse, providing limited food resources from flowering plants.

In contrast, pine forests exhibit a well-developed second tier, which includes plants such as rowan, cherry, and elderberry that actively bloom and attract *P. fieberi* for supplementary nourishment. Forest edges also serve as favourable habitats for *P. fieberi*, where the abundance of species in the upper forest tier does not significantly exceed that found near the grassy layer [56]. The species has been noted even in the vicinity of burned areas and, in small quantities, at fire sites [57,58].

In clearings, the anthophilous behaviour of *Protaetia fieberi* becomes evident as the number of individuals near the ground surface increases significantly [55]. Similar observations were made in Italy, where *Protaetia fieberi* was also captured at heights of 5–6 metres using traps baited with wine and sugar [59]. Rößner [60] also highlighted a greater probability of encountering this species in tree crowns.

Visual observations of *Protaetia fieberi* were conducted across various regions within its distribution area. These observations were primarily limited to manual collection and the use of hand-held sweep nets, which likely contributed to the perceived rarity of the species in the field. Conventional collection methods such as pitfall traps and window traps, which are commonly used in European Russia, proved inadequate for accurately assessing the abundance and condition of *Protaetia fieberi* populations. Additionally, this species was not detected using Malaise traps or pan traps, which is understandable given its rarity in the surface layer and within the first tier of forests (Figure 4).

The most effective method for detecting *Protaetia fieberi* is the use of beer traps at a height of 7 to 12 m. The adults’ feeding behaviour on leaking sap from plants makes these traps particularly successful for capturing the species. Long-term studies have revealed that *Protaetia fieberi* is not as rare as previously thought. For instance, in the Tambov region, beer traps recorded a 46% occurrence rate for *Protaetia fieberi* [61]. Over a five-year period in the Mordovia State Nature Reserve, the species’ occurrence rate was 12.5% [62]. The average occurrence in the Republic of Mordovia over three years was 31% [63]. In the Orenburg region, beer traps showed that this species can constitute up to 30% of the captured beetle species [38]. Additionally, studies conducted in Chuvashia in 2023 reported an incidence rate of approximately 42%. Notably, in Chuvashia, *Protaetia fieberi* was found on a private plot with a few old apple trees and nearby young aspen trees, approximately 2 km from the nearest old oak grove [64].

Thus, when studying *Protaetia fieberi*, the apparent rarity of the species can largely be attributed to the use of less effective research methods. The application of more effective techniques, such as beer traps, reveals that *Protaetia fieberi* is not as rare as previously believed. To accurately assess the conservation status and determine the need for the protection of this species, it is crucial to use beer traps as the primary method for obtaining reliable and comprehensive data.

## 5. Conclusions

According to our data and the literature, *Protaetia fieberi* has been reliably documented in 26 regions of European Russia. The primary limiting factors for its distribution to the north, south, and southeast include the scarcity of suitable biotopes. Within its distribution area, *P. fieberi* prefers deciduous and mixed forests of various types, including anthropogenic environments. The species is notably prevalent in man-made forest ecosystems, such as protective forest belts, old parks, and gardens. In forest ecosystems, *Protaetia fieberi* tends to inhabit the upper tiers of the forest and is seldom found in the surface layer. Conversely, in glades and open areas, the species shows a preference for the surface layer. This distribution pattern correlates with its feeding habits, with adults feeding on leaking sap from tree trunks in forested areas and on flowering plants in open habitats. The seasonal activity of *Protaetia fieberi* peaks from the third decade of June to the second decade of July. The perceived rarity of the species is likely due to the limitations of certain collection methods. Beer traps have proven to be the most effective tool for sampling *Protaetia fieberi*, revealing that the species is more common than previously thought. *Protaetia fieberi* is listed in the Red Book of the Russian Federation and holds a conservation status in several regions [46]. However, recent findings suggest that the species may be more widespread and less rare than previously believed, prompting ongoing discussions about its conservation status.

## Data Availability

Data are contained within the article.
